# Co-Designing a User-Centered Digital Health Tool for Supportive Care Needs of Patients With Brain Tumors and Their Caregivers: Interview Analysis

**DOI:** 10.2196/53690

**Published:** 2025-05-23

**Authors:** Mahima Kalla, Ashleigh Bradford, Verena Schadewaldt, Kara Burns, Sarah C E Bray, Sarah Cain, Heidi McAlpine, Rana S Dhillon, Wendy Chapman, James R Whittle, Katharine J Drummond, Meinir Krishnasamy

**Affiliations:** 1 Centre for Digital Transformation of Health, Faculty of Medicine, Dentistry and Health Sciences, The University of Melbourne, Grattan Street, Parkville, VIC, 3010, Australia, +61 3 8344 4000; 2 Department of Health Services Research and Implementation Science, Peter MacCallum Cancer Centre, Melbourne, VIC, Australia; 3 Department of Neurosurgery, The Royal Melbourne Hospital, Parkville, VIC, Australia; 4 Department of Surgery, Faculty of Medicine, Dentistry and Health Sciences, The University of Melbourne, Parkville, VIC, Australia; 5 Barwon Health, Geelong, VIC, Australia; 6 St Vincent’s Hospital Melbourne, Fitzroy, VIC, Australia; 7 Personalised Oncology Division, Walter and Eliza Hall Institute of Medical Research, Parkville, VIC, Australia; 8 Department of Medical Biology, The University of Melbourne, Parkville, VIC, Australia; 9 Department of Medical Oncology, Peter MacCallum Cancer Centre, Melbourne, VIC, Australia; 10 Department of Nursing, School of Health Sciences, Faculty of Medicine, Dentistry and Health, The University of Melbourne, Parkville, VIC, Australia; 11 Victorian Comprehensive Cancer Centre Alliance, Melbourne, VIC, Australia

**Keywords:** brain cancer, unmet needs, supportive care, psychosocial support, digital health, qualitative research, brain tumor, user-centered, patients, caregivers, interview analysis, quality of life, effectiveness, co-design paradigm, ideas, concepts, emotional support, information sharing, social connectedness, health care professionals

## Abstract

**Background:**

Brain tumors are characterized by the high burden of disease that profoundly impacts the quality of life in patients and their families. Digital health tools hold tremendous potential to enhance supportive care and quality of life for patients with brain tumors and their caregivers.

**Objective:**

This study aims to generate ideas and concepts, through a co-design paradigm, to inform the development of a digital health tool to address the unmet needs of people affected by brain tumors.

**Methods:**

Patients with brain tumors, caregivers, and health professionals from 2 large public tertiary hospitals in Victoria, Australia, were invited to complete a qualitative interview discussing their unmet needs of care. Overall, 35 qualitative interviews focusing on unmet needs and concepts for a digital health tool were conducted with 13 patients, 11 caregivers, and 11 health professionals. Interviews were audio recorded and transcribed, and a 5-step framework analysis approach was used to analyze data.

**Results:**

Four themes of unmet supportive care needs emerged: (1) emotional and psychological, (2) information, (3) physical and practical, and (4) social connectedness. Participants expressed the desire for early and proactive mental health intervention, noted the importance of providing mental health support to caregivers, and emphasized the need for positive stories and affirmative language. From an information perspective, participants noted a sense of information overload, especially at the beginning. They also underscored the variety of information needed on an ongoing basis, including life after treatment, and comprehensive care assistance to maintain quality of life. Participants also described unmet supportive care needs relating to symptom burden, and practical and administrative support to facilitate the logistics of accessing treatment and accomplishing daily life tasks. Finally, they expressed the desire for greater social connectedness and safe spaces to engage with other people in a similar situation. Our findings are consistent with previous research on this subject and were integrated into the development of a web-based platform.

**Conclusions:**

Participants’ perspectives informed the development of content for a web-based digital health platform called “Brain Tumours Online.” The platform comprises three pillars—(1) “LEARN”: a repository of vetted information about a range of biomedical and psychosocial care topics; (2) “CONNECT”: a digital peer support community with a health care professional interface; and (3) “TOOLBOX”: an emerging library of validated digital therapeutics for symptom management.

## Introduction

Despite comprising only 1.2% of all cancers in Australia, the impact of brain tumors on the quality of life is profound [1-3]. Patients with brain tumors experience persistent, distressing, and disabling physical, psychosocial, cognitive, and financial challenges. These challenges are compounded by barriers to connecting and communicating with their treating team, establishing peer support networks, managing their symptoms, and accessing personalized supportive care [4-8]. These issues are exacerbated when patients reside distant from metropolitan, specialist treatment centers [9] without equitable access to comprehensive supportive care services.

Digital health tools hold tremendous potential to revolutionize support that enhances the quality of life for patients with brain tumors and their caregivers within convenient time frames and comfortable environments. Notably, they have the ability to address inadequate cancer services access by overcoming geographical, physical, and psychological barriers, and facilitating treatment access, support, and education [10]. When compared to usual care, health care augmented with digital health interventions has been shown to improve symptom management, reduce distress, decrease unplanned hospitalizations and associated care–related costs, and improve survival and quality of life [11]. While a number of digital supportive care tools for patients with brain tumors have been described [12], there are currently none tailored to the Australian context. In addition, existing tools are limited by a lack of evidence of their effectiveness and impact, development and implementation, and little consumer engagement in their development [7]. Building on our prior research on the quality of life of patients with brain tumors [2,3,13], their patterns of social media use for disease management [8], and needs and expectations from a digital health model of care [7], we engaged with Australian patients with brain tumors, their caregivers, and treating health professionals to co-design a digital health solution to address the unmet supportive care needs of this population. The aim of this study was to generate ideas and concepts to inform the development of a digital health tool to support the needs of Australian patients with brain tumors, their caregivers, and treating health professionals.

## Methods

### Co-Design Approach

Co-designing with patients and end users is widely recognized as critical to the design and development of digital health interventions and is used extensively across a range of physical and mental health conditions [14]. According to Sanders and Stappers [15], co-design may be conducted at various stages of a project life cycle, for example, (1) predesign (to understand lived experiences), (2) generative (to produce ideas), (3) evaluative (to summatively assess solutions), and (4) postdesign (to assess users’ experience of the solution). We adopted a multimodal co-design approach, which included one-on-one interviews, focus groups, workshops, a fortnightly forum called a Design Reference Group, an end-of-life working group to inform design decisions for palliative care resources, and preliminary usability testing of a high-fidelity prototype. This paper reports the findings of one-on-one interviews conducted with patients, informal caregivers, and health care professionals in the project’s “generative” phase. The other co-design activities will be addressed in subsequent research papers and are not included within the scope of this paper.

### Participants and Recruitment Procedure

Three participant cohorts were recruited for this interview study: (1) adult patients (aged over 18 years) with a primary brain tumor, (2) current and bereaved caregivers of adults with a primary brain tumor, and (3) multidisciplinary health care professionals involved in the care of people affected by brain tumors. We excluded patients with secondary brain tumors from systemic cancer and their caregivers. Neurosurgeons were integral to the predesign phase and research team; thus, the generative phase focused on recruiting a variety of other health care professionals, including nursing and allied health staff.

Purposive sampling was used to identify participants with a wide variety of experiences in relation to aspects such as time since diagnosis, age, sex, postcode, tumor types, and types of treatment received. Researchers AB and VS maintained a spreadsheet of participants with the aforementioned salient information and sought to proactively seek out participants from a wide breadth of lived experience.

Participants were recruited from 2 major metropolitan hospitals in the State of Victoria (Australia). Patients were also asked to nominate a caregiver to take part in the study. Bereaved caregivers were also nominated by brain tumor advocacy organizations and consented by a study researcher. Health professional participants (including medical and radiation oncologists, clinical care coordinators, palliative care physicians, neuro-oncology nurses, and other allied health professionals) were identified through research team networks and invited via email.

### Data Collection

The interviews were conducted over a period of 6 months, from October 2021 to March 2022. All consenting participants completed 1 audio-recorded semistructured telephone interview of 20‐60 minutes with a research assistant. Verbal consent was reconfirmed and recorded prior to the commencement of the interview, along with basic demographic information. Semistructured interview guides were developed for each participant cohort (Multimedia Appendices 1-3). Patient and caregiver interviews sought to elicit their lived experiences from diagnosis, their unmet supportive care needs, and their desires and preferences for digital solutions to address identified unmet needs. Health care professional interviews explored their perception of patient and caregiver needs, their experience with the provision of digital-based health care, and their perceptions of the need and preferences for digital health solutions to support patients with brain tumors and their caregivers.

### Data Analysis

The audio recordings from each interview were transcribed verbatim, and the transcripts were inputted into QSR International’s NVivo (version 12.6) for Mac software for coding and qualitative data management. All transcripts were quality-checked and deidentified for analysis by author AB. Data were analyzed using a 5-step framework analysis method [16], as follows:

- Familiarization: Researchers AB and VS read all the interview transcripts to familiarize themselves with the data.
- Identifying a framework: Fitch’s 2008 Cancer Supportive Care Framework [17] was used to develop an a priori thematic framework to guide early data analysis. This is an evidence-informed framework that directly relates to the focus of the study to explore and describe unmet supportive care needs as experienced by people affected by brain tumors and their caregivers. The framework articulates 7 domains of supportive care needs: physical, informational, emotional, psychological, social, spiritual, and practical.
- Indexing: The thematic framework was applied to the data. Three researchers (AB, VS, and M Kalla) first indexed an initial set of 10 interview transcripts using Fitch’s 7 supportive care categories. After this initial application, overlaps were found across the 7 domains, and the analytical framework was revised into 4 thematic categories for this study: emotional and psychological, information, physical and practical, and social connectedness needs. Subsequently, researchers AB and VS indexed all the interview transcripts independently, meeting regularly to ensure consensus around the final relevant themes and subthemes.
- Charting: The indexed data were organized into a manageable chart format to enable within- and cross-case analyses. NVivo’s “Organise” function retrieved charted summaries of the indexed data for each thematic category and individual participant transcripts [18].
- Mapping and interpretation: Finally, mapping and interpretation of the data were conducted through the lens of the 3 pillars of the digital health tool proposed in the original funding proposal, that is, Learn, Connect, and Toolbox. Researchers AB, VS, and M Kalla synthesized the findings into summary presentations, describing how unmet supportive care needs could be addressed through one or more pillars.

### Research Rigor

Our research team was comprised of a multidisciplinary team of academics, health service researchers, clinicians, technology developers, and lived experience experts. The multidisciplinary perspectives afforded by our team members strengthened the conceptualization, analysis, synthesis, and dissemination of our research findings. The majority of the interviews were conducted by the second author (AB), who has experience conducting health services research with patients with cancer and their caregivers using qualitative methods, having worked in a nationally reputed cancer treatment center. A small subset of interviews was also conducted by the first author (M Kalla), who is a digital health and qualitative research expert, and the third author (VS), who has experience as a nursing clinician and qualitative health researcher. This study was overseen by the last author (M Krishnasamy), who is a professor of nursing and qualitative research expert. The overall project was led by author KJD, who is the head of neurosurgery at a major metropolitan hospital in Australia.

To ensure credibility in the analysis of the research findings, 2 researchers (AB and VS) independently analyzed the interview transcripts to generate initial codes. Subsequently, a working group involving authors AB, VS, M Kalla, SCEB, and M Krishnasamy met at fortnightly intervals during the entire data collection and analysis process to compare, refine, synthesize, and establish the final themes. Interviews were conducted until data saturation was reached, and no new topics were discovered, resulting in a total of 35 interviews (13 patients, 11 caregivers, and 11 health care professionals), which is within the suggested range of 9‐17 interviews in qualitative research [19]. Member checking was not conducted with participants to respect the limited available time of patients with brain tumors and caregivers to participate in a research project. Instead, we sense-checked the themes with a lived experience expert (a bereaved caregiver) who was part of our project’s steering group. Finally, the findings were presented to the broader research team, including health care professionals and consumer advocates.

### Ethical Considerations

The study was approved by the Royal Melbourne Hospital’s Human Research Ethics Committee (HREC/77238/MH-2021). The ethics committee is accredited with Australia’s National Health and Medical Research Council and is operated in accordance with the National Statement on Ethical Conduct in Human Research. The data presented in this paper are the primary data that were collected as part of this ethical approval. Potential participants were emailed an information and consent form to read and sign to agree to be interviewed. To ensure privacy and confidentiality, all study data were deidentified prior to analysis. Author AB reviewed all interview transcripts in their entirety to ensure that they had been fully deidentified prior to the commencement of analysis. Identifiable participant information was securely stored with password protection and accessible only to researchers authorized under the ethical approval.

Interviews were conducted over the telephone, with costs for the phone calls borne by the research team. Participants were not required to travel, and the interviews were conducted at a time suitable for them. Therefore, participants did not face any financial burden from participating in the study. Thus, reimbursement was not provided for their voluntary participation. The aforementioned arrangements for participant involvement were approved by the ethics committee, as articulated in the research study protocol submitted at the time of application. As part of the ethics approval, our team was also required to submit annual progress reports and a final report, as well as notify the committee of any adverse events. These reports were provided to the committee in a timely manner. There were no adverse events reported from the conduct of this research.

## Results

### Overview

A total of 35 participants (13 patients, 11 caregivers, and 11 health care professionals) were recruited (Table 1).

**Figure 1.**
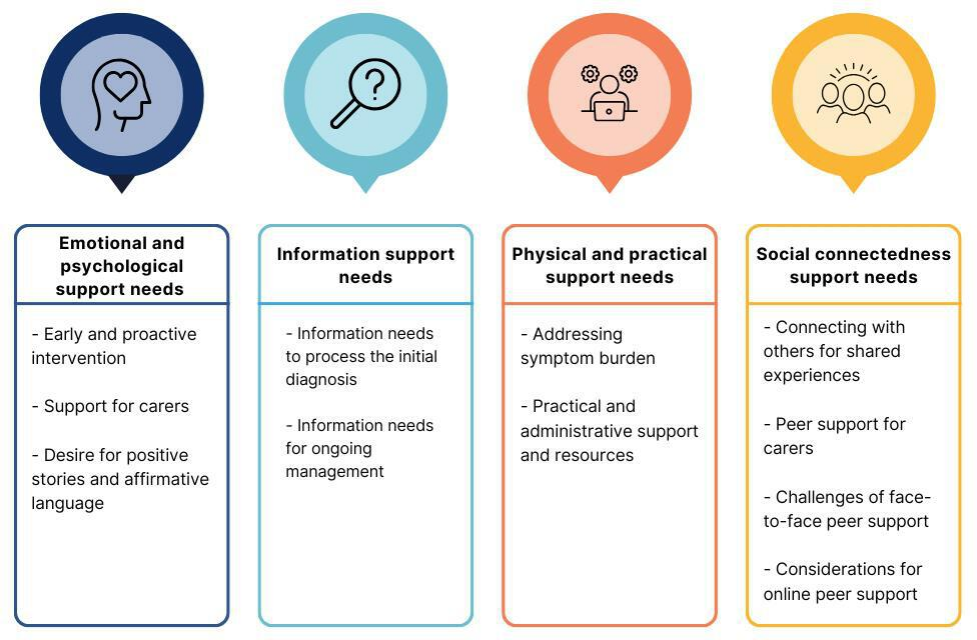
Overview of emergent themes and subthemes.

The emergent themes and their subthemes are presented in Figure 1. Detailed and illustrative quotes from each of the 4 support needs domains—emotional and psychological, information, physical and practical, and social connectedness—are presented. All data fit within these themes on completion of the iterative process.

**Table 1.** Overview of participant demographics (N=35).

Demographic	Patients (n=13)	Caregivers (n=11)	Health professionals (n=11)
Age (years), mean (range)	42.1 (22‐67)	55 (32‐86)	47.1 (40‐60)
Sex, n (%)			
Male	5 (39)	2 (18)	3 (27)
Female	8 (62)	9 (82)	8 (73)
Location, n (%)			
Metropolitan	9 (69)	4 (36)	8 (73)
Regional or remote	4 (31)	4 (36)	3 (27)
Unanswered	0 (0)	3 (27)	0 (0)
Tumor type, n (%)			
Low-grade glioma	5 (39)	N/A^a^	N/A
High-grade glioma	5 (39)	N/A	N/A
Rare brain cancer	3 (23)	N/A	N/A
Relationship to the patient, n (%)			
Spouse	N/A	6 (55)	N/A
Parent	N/A	4 (36)	N/A
Child	N/A	1 (9)	N/A
Caregiver status, n (%)			
Current	N/A	9 (82)	N/A
Bereaved	N/A	2 (18)	N/A
Role, n (%)			
Neuro-oncology nurse	N/A	N/A	3 (27)
Clinical care coordinator	N/A	N/A	2 (18)
Medical oncologist	N/A	N/A	2 (18)
Neuropsychologist	N/A	N/A	1 (9)
Palliative care physician	N/A	N/A	1 (9)
Exercise physiologist	N/A	N/A	1 (9)
Radiation oncologist	N/A	N/A	1 (9)

a N/A: not available.

### Theme 1: Emotional and Psychological Support Needs

Within the theme of patient and caregiver emotional and psychological support needs, 3 subthemes highlighted the need for early and proactive psychological intervention, the importance of emotional support for caregivers, and the desire for positive stories and affirmative language.

#### Early and Proactive Intervention

Participants reported delayed help-seeking for mental health assistance due to a lack of awareness of relevant services and the benefits of intervention. They described frequent reliance on informal mental health support from family and friends, or those with similar experience. Some participants delayed addressing mental health needs until after treatment. Almost all participants wished they had been encouraged by their treating team to access mental health services or provided with links to resources and tools at diagnosis.

*I didn’t start seeing a psychologist until after treatment which isn’t good...I wish someone kind of forced me to see one, not forced...like just did a referral for me to see one...I did like a year of treatment and...you’re trusting your family [but] there’s only so much they can really do...Before treatment would have been good...Once I was diagnosed, I wish I had saw [sic] someone.*

[Patient 3, female, 22 years]

Health care professionals reported varied levels of proactiveness for mental health service referrals. Lack of referral was often driven by insufficient clarity regarding the best service or intervention for their patient’s specific needs. Often, health care professionals deferred to brain tumor advocacy organization services and web-based resources.

*I have...people saying...I’m quite distressed...and then me...going through this list...so we’ve done the referral to Cancer Council, you’re on a wait list for a counselling session...What exactly is the issue, can it be worked through with a counsellor, is it more affecting...do we need psychology support, or do we need some...medication intervention...do I need psychiatry...I think just having that information a bit more available.*

[Health care professional 10, neuro-oncology nurse consultant, female, 39 years]

#### Support for Caregivers

Participants highlighted the importance of mental health support for caregivers, with mental health discussions often focused on the patient and inadequate recognition of caregivers’ needs.

*I think as carers you don’t recognise...oh actually your life is going to be significantly impacted in the future and...right here and now you actually need to look after yourself as much as you can, to be the best carer that you can [be].*

[Caregiver 2, female, 37 years]

*I was offered counselling...I reckon it should be available to both parties...I feel that my partner...she was going through more than me.*

[Patient 6, male, 31 years]

#### Desire for Positive Stories and Affirmative Language

Participants expressed the need for information and lived experience stories, framed positively and fostering a sense of hope. Some participants actively sought people with brain tumors on social media who were living fulfilling lives, as it gave them hope for their own future.

*Just to really emphasise that your life isn’t over, and you can still have a normal life after it...I found there’s a few people on Instagram that had been diagnosed with it and they’re still out there...really doing well.*

[Caregiver 4, female, 32 years]

Affirmative explanations of complex medical treatments and procedures were useful in creating a greater understanding of the treatment plan and reducing fear.

*A lot of people are actually frightened...having brain surgery whilst you’re awake, is such a morbid type of experience, where I found it exactly the opposite...It’s hard to describe the experience that I went through, but being able to listen to what’s going on whilst it’s happening...oh we’re removing some of the tumour now, having all that conversation...I found that [I was] more in control...more knowledge, more knowing...everything that was going on.*

[Patient 7, male, 50 years]

### Theme 2: Information Support Needs

Participants described significant initial information needed to process their diagnosis and ongoing information needed to manage the physical, social, and psychological impacts of a brain tumor on their lives and that of their families.

#### Information Needs to Process the Initial Diagnosis

Information requirements were greatest at diagnosis and reported a sense of overwhelm with “information overload.” Patients and caregivers reported difficulty understanding the information presented by their health care professionals and expressed a strong need for tailored, personalized information, appropriate for the type and location of tumor and patient age and sex. Similarly, health care professionals recognized that deconstructing medical jargon while providing the appropriate breadth of information is crucial to support patients and their caregivers in decision-making around treatment options.

*I think you’ve got to find a balance between not overloading people with information but giving them access to resources and that’s never easy.*

[Health care professional 8, medical oncologist, male, 55 years]

At diagnosis, patients and caregivers described turning to internet searches to learn more about the tumor, treatment options (including clinical trials and integrative therapies), life expectancy, and survival statistics. Many participants reported that in these early stages, they struggled to discern credible information and found “Dr Google” overwhelming.

*I still wanted to know...as much information as I could about...my diagnosis...but I needed accurate information and that’s where I say the internet didn’t really supply me that accurate information about my particular case.*

[Patient 7, male, 50 years]

#### Information Needs for Ongoing Management

Patients and their caregivers described an ongoing need for information about life after treatment, the impacts of the illness and its treatment, and assistance to maintain quality of life.

*Help processing what the treatment plan looks like...and knowing where to go for help and answering questions. Not only about diagnosis but also about treatment once they’re discharged...The discharge happens pretty quickly from surgery, usually...six to nine days or sometimes sooner.*

[Health care professional 5, cancer care coordinator, female, 51 years]

Additionally, patients and caregivers expressed the need for on-demand information in a consolidated space and the ability to learn from experts from the comfort of their homes.

*Video would be good...hearing from experts and doctors about things would be really helpful...And maybe even a Q&A session where you can ask questions, and they can answer...interactively.*

[Caregiver 7, female, 60 years]

### Theme 3: Physical and Practical Support Needs

Participants described unmet supportive care needs relating to symptom burden, practical and administrative support to facilitate the logistics of accessing treatment, and accomplishing daily life tasks.

#### Addressing Symptom Burden

Participants described a variety of symptoms associated with the brain tumor or its treatment, which significantly impacted patient quality of life and caregiver burden. Participants expressed a strong desire for access to resources and interventions to support symptom management, including sleep disturbance, fatigue, cognitive dysfunction, poor concentration, imbalance, and incoordination. Participants indicated that symptoms were clustered, for example, an interaction of sleep disturbance and fatigue.

*If there was something to help with sleep, I would definitely jump...that would be one of the biggest things...I basically always went to the GP about...I would go to say I can’t sleep, I just get...rundown...I can’t sleep, I can’t sleep, I’m tired...*

[Patient 1, male, 53 years]

Seizures were considered particularly burdensome and “frightening” with management options often not well understood by patients or caregivers.

*Managing seizures and what to do in, in the event of a seizure...I mean not everyone needs to come to hospital if they have a seizure, it depends on...what type...Partial seizures...you can pretty much manage at home. And then...talk to someone the next day or go through the GP...there’s certain steps that you could take. I think that would be a really good [pause] thing to put in...if you’re uploading information [on the platform].*

[Health care professional 5, cancer care coordinator, female, 51 years]

We asked about the role of self-monitoring by patients for symptom management, with participants presenting mixed responses. Some health care professionals, particularly in allied health and palliative care contexts, used symptom monitoring to support patients. Nevertheless, participants agreed that symptom monitoring regimes, whether digital or paper-based, would need to be considered contextually within broader clinical workflows to be useful or effective.

*I guess the key thing would be somebody taking responsibility for that and...following up where concerning symptoms have been presented...I don’t know that just collecting symptoms for the sake of collecting symptoms is going to be particularly useful. You’ve got to have somebody who’s going to be reviewing that information and...acting upon it.*

[Health care professional 9, radiation oncologist, female, 51 years]

#### Practical and Administrative Support and Resources

Participants expressed the need for support and resources to manage practical, logistical, or administrative challenges. Information to navigate transport, permission to continue or return to driving, government welfare and advocacy agency support, and accommodation during treatment and management of insurance were commonly difficult to obtain.

*The thing that really frustrated me is every time I’d try and talk to someone about...things like financial information...life insurance...income protection...just to try and get some information and help with trying to sort through...how I can access [it].*

[Patient 1, male, 53 years]

Participants also emphasized the particular need for practical, logistical, and administrative support for patients and caregivers in rural or regional areas.

*We need to keep in mind the...regional family as well because...there are types of cancer that can’t be treated in...regional areas. They need to be treated at a specialist centre. So...demands placed on that around travel...the financial impacts of that...paying for accommodation, parking, travel all those sorts of things. There are subsidies and things that people can get access to but that does have impacts that are ongoing...taking time off work...all those sorts of things are quite huge.*

[Health care professional 1, exercise physiologist, male, 42 years]

When asked about telehealth, all patients and caregivers reported having used telehealth and found it time-saving and convenient. However, for important appointments, a face-to-face conversation was preferred.

### Theme 4: Social Connectedness Support Needs

Patients and caregivers expressed the need for social connectedness and suggested these may be met by a digital platform. Four subthemes emerged: the importance of connecting with others for shared experiences, the significance of peer support for caregivers, challenges and lessons learned from face-to-face peer support programs, and considerations for creating safe and beneficial digital peer support communities.

#### Connecting With Others for Shared Experiences

Participants noted that a brain tumor can be alienating, with feelings of isolation further compounded for those in rural or regional areas. Thus, opportunities to connect with others with similar experiences can offer solace.

*When you find someone who you can relate to and they’ve got a level head, it just puts your mind at ease.*

[Patient 6, male, 32 years]

Participants noted that in addition to helping cope with stressors, peer support could also normalize living with this illness.

*This is the absence that we have in brain cancers and tumours...you can see the hard, the scary facts, but...it is really to understand what next, who else is out there like me, who else is young and also about to get married, or just about to have a baby, or just got the promotion at work or...I feel like there’s a lot of people that sit in this category.*

[Caregiver 2, female, 37 years]

#### Peer Support for Caregivers

Participants also highlighted the need for social connectedness and peer support for caregivers and noted that digital solutions could offer a helpful on-demand avenue for busy caregivers.

*Online [peer support] is actually like a great entry point...You’re so consumed by...needing to do everything for that person...I can’t remember how many times I would’ve been awake at 2 am in the morning or something because my brain was racing, and being able to may be connect with somebody or...something like that, not necessarily to chat right there in that moment.*

[Caregiver 2, female, 37 years]

Participants also emphasized the importance of peer support for caregivers in all situations, including those currently undertaking caring duties, as well as bereaved caregivers. Some participants had found established grief support groups, but not for current caregivers, while other participants reported a lack of formalized support structures for bereaved caregivers.

*Our focus is on the patient and of course we support the partner while they’re coming to the centre. But once that patient’s passed away our involvement sort of ends, and I think these partners are left...often they’re very fatigued because I think [for] the carers of these types of patients...the carer burden is huge.*

[Health care professional 4, nurse, female, 46 years]

#### Challenges of Face-to-Face Peer Support

Participants expressed that despite their desire for social connection with others with similar experiences, they had either not been able to find an in-person support group that was the right fit for them, or they had found the support networks unhelpful or not relatable with members of varying experiences, ages, sexes, or life stages. Additionally, in-person support groups could be confronting, and digital peer support was considered a helpful alternative.

*She didn’t want to go to a room and sit in a chair around in a circle and tell her story...Some of those support groups can be a bit too full on. So that’s why something that isn’t quite as full on like an app or online story or...live chat, something...you can still...perhaps talk to someone, so you’re...not in a room full of strangers...But something in between they can just have on their smart phone or computer and just send off an enquiry and if that’s all you need at the time, then that’s fine.*

[Caregiver 1, male, 37 years]

Health care professionals with experience in running in-person support groups also highlighted the logistical challenges of facilitating face-to-face groups. They reported high attrition rates, emphasized the need for distress protocols in the event that a participant is emotionally affected, and noted the complexities of running in-person support groups involving people with varying prognoses.

*If you’re meeting in person you’ve got to have reasonable leaders...Normally the social worker would do it with me...because if someone gets upset, you’ve got to have enough people to take them out and talk to them separately as well...I think the support groups do help but...you have to be careful, and you can’t mix the low grade with the high grade. That’s another problem because...the high grade have very immediate needs.*

[Health care professional 7, care coordinator, female, 72 years]

#### Considerations for Digital Peer Support

Challenges to create a safe, relatable, and where possible, customizable web-based peer support platform were recognized as important considerations. Participants commented on the importance of regulation and moderation in digital peer communities. They expressed concerns over harsh language, individuals with domineering personalities and negative responses, or harmful opinions being promoted within an already vulnerable group.

*You have different...personalities, and I’d be worried if you did an online group that some people might be afraid to speak up because there could be other people that are quite boisterous.*

[Caregiver 3, female, 49 years]

Participants emphasized the need for control over the visibility of information and the social media posts of others. They reported that social media news feeds can be jarring and compound emotional distress.

*My sister joined this Facebook group for...brain tumour and she...added me along. I’ve sort of been a little bit up and down about that...because it just comes up on my news feed and then you know some...things that I read...is okay, but then...some things that I read that aren’t so great, I sort of tune off a little bit you know.*

[Patient 9, female, 38 years]

Similar to in-person peer support groups, digital peer communities need to consider the implications of varying prognoses due to different tumor types. When asked about different digital peer support formats, such as a “buddy” program, participants expressed concerns that those who bond with other unwell individuals may open themselves to grief if their “online buddy” dies or becomes too unwell to participate.

*What happens when something happens to my buddy, you know, like, what does that gonna traumatize? [sic]...I don’t know.*

[Patient 12, female, 39 years]

### Integration of Emergent Findings Into the Platform

The participants’ insights were used to inform the development of content for our web-based platform now called “Brain Tumours Online.” The Brain Tumours Online platform features three key elements or “pillars”: (1) a repository of vetted, evidence-based information about a myriad of health, social care, and administrative supports available to patients and their caregivers (“LEARN”); (2) a digital peer support community to enable connections with other patients, caregivers, and health care professionals (“CONNECT”); and (3) an emerging library of validated digital symptom management therapeutic solutions (“TOOLBOX”). Herein, we describe how participants’ insights were synthesized and addressed by the various components or “pillars” of the Brain Tumours Online platform.

Based on the emotional and psychological and social connectedness themes, participants’ perspectives on face-to-face and digital peer support informed the design of our web-based peer community under the CONNECT pillar. Our digital peer community mitigates the access barriers (eg, time and geographical constraints, privacy concerns) for people who noted challenges with in-person support groups. Our platform’s CONNECT pillar provides a range of avenues for sharing stories, experiences, and knowledge, for example, topic-specific chat forums, users’ individual posts, and digital webinars. The CONNECT pillar also provides an avenue for sharing other patients’ and caregivers’ past hopeful stories, addressing the unmet need for positive stories that foster a sense of hope. The participants’ preference for a safe space was also translated into the implementation of a moderator group, which includes moderator-trained patients, caregivers, and health care professionals. The CONNECT pillar also provides health care professional interface, including via digital webinars, and in their capacity as community moderators, to answer any questions on chat forums and vet information.

Within the information theme*,* participants expressed the unmet need for trusted and bespoke medical and social care information. Our platform’s LEARN pillar provides a consolidated repository of brain tumor expert-endorsed information that is relevant to the Australian context, with signposting to specific tumor types, life stages, and personal situations. Examples of information available on the platform range from social care options for childcare, treatment-related travel subsidies for patients in remote and regional areas, eligibility for motor vehicle driving, and what to expect in various biomedical treatment options. The LEARN pillar contains a combination of curated existing external (trustworthy and vetted) resources and new resources freshly developed for our digital health platform where an existing suitable resource was not already available.

Within the physical and practical theme, participants noted their unmet needs for resources and interventions to manage symptom burden. These needs are addressed by the Brain Tumours Online platform’s TOOLBOX pillar. The TOOLBOX is intended to become a growing library of validated digital therapeutic tools for symptom management. The first tool that is currently available in the TOOLBOX is “Somryst,” a Food and Drug Administration–approved digital therapeutic tool for chronic insomnia. Additional tools will be incorporated into the platform TOOLBOX in due course, in response to our user community’s needs.

## Discussion

### Principal Findings

In this study, we set out to generate ideas and concepts to inform the development of a digital health tool to support the unmet needs of Australian patients with brain tumors, their caregivers, and treating health professionals. The data revealed 4 themes of unmet support needs that could be addressed by a digital health tool or platform: emotional and psychological, information, physical and practical, and social connectedness. Participants expressed the desire for early and proactive mental health intervention, noted the importance of providing mental health support to caregivers, and emphasized the need for positive stories and affirmative language. From an information perspective, participants noted a sense of information overload, especially at the beginning. They also underscored the variety of information needed on an ongoing basis, including life after treatment, and comprehensive care assistance to maintain quality of life. Participants also described unmet supportive care needs relating to symptom burden, practical and administrative support to facilitate the logistics of accessing treatment, and accomplishing daily life tasks such as work and study. Finally, they expressed the desire for greater social connectedness and safe spaces to engage with other people in a similar situation.

### Comparison With Prior Work

The unmet supportive care needs identified in our study echo some of the previous literature published in this area. For example, Janda et al [20] conducted a qualitative exploration of the supportive care needs of patients with brain tumors and their caregivers. They identified the need for greater practical support (eg, support with financial issues and dealing with government agencies), the need for information and coping with uncertainty, as well as support to deal with social isolation. They found that technology could be a helpful avenue for patients in obtaining information and supporting caregivers. Our Brain Tumours Online platform seeks to provide a digital peer support mechanism to help mitigate social isolation and bridge information access gaps.

Indeed, the literature also emphasizes the changing needs of patients and caregivers over time. Previous studies indicate that information and mental health support needs are often greatest at the start and can change over time [4]. Thus, past literature emphasizes the need for bespoke and adaptable information and supportive care resources that can assist patients and caregivers in accordance with their evolving needs over time. Furthermore, past studies also indicate patients and caregivers wish to stay abreast of the latest developments in research and treatment of brain tumors [21]. To this end, our Brain Tumours Online platform seeks to serve as a living resource that can support patients and caregivers at different stages of diagnosis, treatment, and posttreatment living, and present a synthesis of the latest developments in this field.

Based on past research, it appears that a key factor in the mitigation of patients’ and their caregivers’ unmet needs was the awareness of available psychosocial support services and consequently their service use [22]. Therefore, a key aim of the Brain Tumours Online platform will be to provide a consolidated set of evidence-based resources on a variety of subjects ranging from availing social care services through government agencies to managing physical symptoms through allied health supports.

While our research findings are consistent with the past literature, our team recognized that existing tools for cancer care have often had little direct consumer engagement in their conceptualization, development, and implementation [7]. Thus, our qualitative exploration reported in this study was essential to validate previous literature and ensure that the emergent Brain Tumours Online platform meets the needs of Australian patients with brain tumors and their caregivers.

### Limitations

First, it should be noted that some of the unmet needs expressed by our study’s participants related to more systemic medical practice challenges, and could not be feasibly addressed through our digital solution, for example, the timeliness of referrals to mental health services. Nevertheless, by providing access to a supportive community, a vetted repository of trusted medical and psychosocial information, and easily accessible digital therapeutics, the Brain Tumours Online platform seeks to bridge comprehensive care and access gaps for a vulnerable patient and caregiver community. Second, we note that this study was aimed at adult participants only. Due to human research ethical constraints, and the scope of our study, pediatric patients were not included. The unmet needs of pediatric patients will need to be explored in a future program of work.

Finally, we note that there were also some limitations associated with our methodological approach. To minimize participant burden, particularly for patients and caregivers, we conducted interviews over the telephone. However, the conduct of interviews over the telephone, as opposed to in-person or via video call, meant that there was limited to no opportunity for identifying nonverbal communication such as body language and facial cues, which can be invaluable in qualitative lived experience research. Similarly, the opportunity for building personal rapport between the researcher and participant is limited in a telephone interview as compared to an in-person interview, which can in turn impact how freely participants share their personal experiences.

### Conclusions

To our knowledge, when we began this project, there were no comprehensive digital health supportive care solutions for Australian patients with brain tumors and their caregivers. Building on our prior research on the quality of life of patients with brain tumors [2,3,13], their patterns of social media use for disease management [8], and needs and expectations from a digital health model of care [7], we engaged with Australian patients with brain tumors, their caregivers, and treating health professionals to co-design a digital health solution to address the unmet supportive care needs of this population. Participants’ insights were distilled to develop content for a web-based supportive care platform called “Brain Tumours Online.” The platform comprises three pillars—(1) LEARN: a repository of vetted information about a range of biomedical and psychosocial care topics; (2) CONNECT: a digital peer support community with a health care professional interface; and (3) TOOLBOX: an emerging library of validated digital therapeutics for symptom management.

## Supplementary material

10.2196/53690 Multimedia Appendix 1 Interview questions for health professional cohort.

10.2196/53690 Multimedia Appendix 2 Interview questions for carer cohort.

10.2196/53690 Multimedia Appendix 3 Interview questions for patient cohort.

## Abbreviations

HREC: Human Research Ethics Committee
