# Supplementary material for: Co-Designing a User-Centered Digital Health Tool for Supportive Care Needs of Patients With Brain Tumors and Their Caregivers: Interview Analysis
Source: JMIR Cancer. 2025 May 23;11:e53690. doi: 10.2196/53690 (PMC12124322; doi:10.2196/53690)
Supplement: Multimedia Appendix 1 [file cancer-v11-e53690-s001.docx]

**Interviewer: Thank you for your time today.**

The purpose of the interview is to ask about your views of the challenges and issues experienced by people affected by brain cancer, and whether you feel a technology-enhanced survivorship model of care could benefit this group of people.

Also, I would like to hear your views about whether a technology-enhanced resource [online platform] could value add to the care you provide to the people you care for.

*I am going to turn on the audio-recorder now, and so please can you state your name and confirm that you are happy to take part in the interview. If at any time you would like to stop this interview, please let me know.*

I’m now going to start with some questions about your experience of providing treatment and care for people affected by brain cancer.

# Semi- structured Interview questions:

# From your experience what do you see, as or believe to be, the most challenging aspects of a brain cancer diagnosis for the patients and family members you care for?

# *For example, their understanding of brain cancer at time of diagnosis; prognosis and discussions about palliative care; the symptoms or side effects they experience; their need for information, emotional support etc.*

# Do you know whether any of your patients access brain cancer support groups?

# *If yes, is your impression that these are helpful?*

# *Do you think that family members/carers have any particular challenges in getting or accessing support?*

1. **How easy do you think it is for your patients or family members/carers to contact a member of their treating team**, if they want to speak with someone about a concern?

*What happens in your Centre?*

*Do you think that patients or carers trying to contact a member of their treating team at your centre face any particular challenges?*

*Do you think there are any particular gaps in services or process available to them in terms of communicating with the hospital?*

1. **Do you think that the online platform being developed through this study could help with connectivity between patients/family members and their health care teams?**

*If yes, in what way? If no, why not?*

*If you feel it could be helpful, are there barriers or enablers in your Centre to making an online platform part of the model of care for patients/carers?*

# Are there any particular aspects of care or treatment that you feel need be made easier for teams treating people with brain cancer?

# *For example, co-ordination of care; models of follow up or survivorship, or the members of your team, e.g. access to a nurse specialist, social worker, psychologist, exercise physiologist*

# *How could an online platform help with these?*

1. **One feature we are proposing for the online resource is the ability for patients to record their symptoms via PROMs, that would be visible to the treating team.**

# *In your experience, do patients or carers have difficulty quantifying or describing the problems they experience?*

*Do you think this would be useful to you and your team? If yes, how; If no, why?*

Would you want this feature to be available during treatment and/or post-discharge?

*Where would data get reported, to whom and when?*

*Are there any particular symptoms/problems you would want to be alerted about?*

*Do you think this kind of feature could prevent any patient or system level issues?
If yes, what do you think they could be?*

*How often would you think patients would need to post data? Once a day, a week, etc.*

*If this was available in your Centre now, what resources would you need to make it work?*

1. **Do you currently recommend any apps or websites to your patients to help them manage any symptoms or side effects- or to get supportive information or advice?**

*If yes, can you tell me which ones you recommend and why?*

*If no, why not?*

1. **Are you familiar with any online interventions to manage problems such as insomnia or anxiety etc?**

*If yes- which ones, and do you recommend any of these to your patients?*

*If no, you do not recommend them, can you explain why?
If you are not aware, is this kind of intervention (if proven/evidence based) something you would consider suggesting for your patients?*

# Are there any particular issues that you think could be included in a frequently asked questions resource on the online platform?

# *Are there any other problems (in addition to the ones you would include in a FAQ list) that you think patients and their families want/need more support with?*

# *Do you think an online platform could help with these issues? If yes, in what way?*

#

# What’s your experience of using telehealth been?

# *Do you feel this has been an effective mode of communicating with your patients?*

# *If it was possible to connect with your patients for parts of their care and follow up via a website or an online platform would you use it?*

# Is there anything you think is important for us to consider as we develop the online survivorship resource?

**When we first spoke about the study, I mentioned the opportunity to be involved in a future workshop. Are you happy to be contacted to see whether you would be available to take part in a workshop over the next few weeks?**

**Yes**

**No**

**Thank you for sharing your experiences and for your time and interest in participating in the interview.**
