# Supplementary material for: Co-Designing a User-Centered Digital Health Tool for Supportive Care Needs of Patients With Brain Tumors and Their Caregivers: Interview Analysis
Source: JMIR Cancer. 2025 May 23;11:e53690. doi: 10.2196/53690 (PMC12124322; doi:10.2196/53690)
Supplement: Multimedia Appendix 2 [file cancer-v11-e53690-s002.docx]

**Semi- structured Interview questions:**

Interviewer: Thank you very much for agreeing to take part in the interview today.

Do you have any questions of me before we start? (if yes- explore and respond)

I am going to turn on the audio-recorder now, and so please can you state your name and confirm that you are happy to take part in the interview.

Thank you. I’m going to start with some questions about the experiences of your family member/friend living with brain cancer.

# Before your family member /friend was diagnosed, what was your understanding of brain cancer?

What is your understanding now?

1. **We are interested to learn about the experiences of people supporting someone affected by/living with brain cancer?**

What things have been hard for your family member/friend and you (the family) since this diagnosis? – including the time up to when the diagnosis was confirmed.

Were you as a family member/carer involved in decisions about treatment?

1. **Some** **people tell us it’s difficult to find out about or access support groups or organisations for people who have brain cancer or their family members or friends. What has been your experience?**

*How did you find the group(s)?*

*If you have experienced problems, what particular challenges of gaps have you experienced?*

*Have you found or access a support group?
Has that/those been helpful? If Yes or no, why?*

*What were you hoping to get or have addressed by joining a group(s)?*

1. **Has your family member/friend had any communication with a brain tumour support group or organization (online or in person)?**

**If yes** - How did they find the support organization?

Were there particular things about the group that you found helpful or unhelpful? What were those?

1. **Who or what has supported you as a carer?**

*Do you think it is important that patients and their carers are able to connect with people in the same situation?*

1. **If you were going to be part of an online peer support group, what features or supports would you like to see as part of the online group?**
2. **Some people say they like to use apps or websites. Do you or your family member/friend use apps or websites for information or support related to brain cancer?
   If yes,** what do they like/don’t like about them?

What are the kinds of features or content that work for them? (for example, videos from other people going through the same thing; hearing experts give advice or information: knowing about new treatments or clinical trials; blogs, Q&A etc)

**If no,** If they don’t use apps or websites, why is that?

What kinds of things would you see as helpful from your experience of living with someone affected by brain cancer– for example, information about treatment, where to get expert advice?

# We know from what patients tell us, that brain cancer and its treatments cause symptoms and side effects that can be challenging and upsetting

1. **What symptoms/side effects has your family member/friend been experiencing?**

**How have these symptoms been managed?**

What things have been the most troublesome/problematic?

How have you and/or they managed these?

Does you or your family member/friend keep a record of symptoms so that they can be shared with the treating team?

**If yes**, do you/they record these on a daily or weekly or monthly basis?

Do you/they find this helpful- and if so, why?

When you or your family member/friend have an appointment with the hospital, is it hard to describe or quantify the impact of symptoms or problems to the team?

If it was possible to keep a record of these in an online platform, would that be helpful to you and/your family member/friend? If yes, why?

How often would it be useful to have feedback on the information recorded?

If that information went straight to the treating team, would that be helpful to you?

If yes, how would that be helpful?

**For some symptoms, such as tiredness or being unable to sleep, anxiety or fear of cancer recurrence, there are online programs that can help someone cope with or manage these.**

1. **If your family member/friend had these symptoms, do you think they would try an online program to manage them**?
   **If yes,** can you tell me why? **If no,** can you tell me why?

If yes, are there particular things they would need from the online program that would make them use it (e.g. – the accent of the person delivering it, how long it went on for, the ability to report back how you were going; having feedback from someone)

If you experienced these kinds of problems, would you be interested in having access to online programs?

What would you hope to get or achieve from these kinds of programs?

1. **Has your family member/friend had to use telehealth much since the pandemic started – or were they using it before?**

*By telehealth we mean more than a phone conversation, eg a video consultation.***What’s your experience of telehealth been?**

If it was possible for you and your family member/friend to connect with their treating team for parts of their care and follow up via a website or an online platform, do you think you and/or they would use it?

**If yes,** why would they be happy to do this- what benefits can you see?
**If no,** can you tell me why this wouldn’t work?

1. **We know that peoples’ needs for information and support can change over time. Can you think about things that you and your family member/friend may need looking forward?**
2. **Is there anything else that you would like to tell me about your experience or support needs before we finish today?**

Conclusion: Thank you for sharing your experiences and for your time and interest in participating in the interview.
