# Supplementary material for: Co-Designing a User-Centered Digital Health Tool for Supportive Care Needs of Patients With Brain Tumors and Their Caregivers: Interview Analysis
Source: JMIR Cancer. 2025 May 23;11:e53690. doi: 10.2196/53690 (PMC12124322; doi:10.2196/53690)
Supplement: Multimedia Appendix 3 [file cancer-v11-e53690-s003.docx]

**Semi- structured Interview questions:**

**Interviewer:** Thank you very much for agreeing to take part in the interview today.

The information you share with me will help the project team better understand peoples’ experiences of a diagnosis of brain cancer and help us develop an online (website or app) resource to support people during and after treatment.

Do you have any questions before we start? (if yes- explore and respond)

- I am going to turn on the audio-recorder now, and so please can you state your name and confirm that you are happy to take part in the interview. If at any time you would like to stop this interview, please let me know. *Any information you give us during the interview will be kept confidential.*

Thank you. I’m going to start with some questions about how you have been since being told about your diagnosis.

# Can you tell me about what had been happening for you in the time before your diagnosis and how things were for you in those first few weeks after being told?

*Were you involved in discussions about treatment and treatment decision-making?*

*Did you have an opportunity to be involved in discussions about the cancer or treatment decision with members of the health care team, for example, a doctor or nurse?*

*Do you feel you were given enough time by members of the health care team to ask questions?*

1. **Did you feel that your support needs (for example, your need for emotional support, or your need for information about your disease and treatment, or financial support, or how to talk to your family or friends about your illness,) were met at that time?**

*If yes- who helped you at that time and why was the help they gave you useful?*

*If no - Looking back is there anything you think would have been helpful at that time that you didn’t have? Why would that have been helpful?*

*Was there anything that happened at this time that was particularly helpful? Can you tell me what that was and why?*

*And what about as you went on through treatment or follow up care? Did you feel your support needs were met during these times?*

1. **Do you find it easy to get in touch with your treating team if you have any questions or concerns?**

*If yes, what makes it easy?
If no, what are the blocks or challenges to connecting with them?*

1. **What things do you wish you’d been told about sooner or had access to sooner?**

*How would those things have helped you or your family/friends?*

1. **Have there been things you have started doing (or carried on doing) for yourself that have been helpful, since you were diagnosed?** *E.g. connecting with family/friends, using websites or support groups for information and support, complementary medicines, counselling, help with cleaning, garden, etc.*

*If you have started doing anything new or kept doing things you’ve always enjoyed- how have these been helpful?*

*If you haven’t done anything new or have had to stop doing thigs you enjoy- is there anything you can think of that would have helped you to do these things?*

1. **Some people tell us it’s difficult to find out about or access support groups or organisations for people who have brain cancer or their family members or friends. What has been your experience?**

*Do you think it is important that patients and their carers are able to connect with people in the same situation?*

*If yes, why is that? If no, what would be helpful?*

1. **Have you had any communication with a brain tumour support group or organization (online or in person)?**

*If yes - How did you find (locate) the support organization? Were there particular things about the group that you found (or still find) helpful or unhelpful? What were/are these?*

*If no, is this because you didn’t want to access a support group?*

*Do you get help or support in other ways?*

1. **If you were going to be part of an online peer support group, what features would you like to see as part of the online group?** *For example, a closed group - that is it’s for just people in the same situation who are invited into the group, to share information or experiences*
2. **If you did join a support group, who do you think should lead or facilitate the meetings?** *Should this be a health professional or a person with cancer ?*

*Can you tell me why you think it should be a health professional/person with cancer?*

*Do you think there are disadvantages or benefits to not having a health professional lead the group? If so, why?*

1. **If there was an option of being linked with a “buddy” – that is someone who has been through an experience similar to yours, and who can provide one-one support, do you think this would be helpful to you?**

*If yes – why would this be helpful?*

*If no, what other kind(s) of one-one support would be helpful, why?*

1. **Some people say they like to use apps or websites. Do you use apps or websites for information or to access support related to your cancer?**

*If yes, what are the kinds of features or content in an app or website that you like and that work for you? (for example, videos from other people going through the same thing; hearing experts give advice or information: knowing about new treatments or clinical trials; community resources, etc).*

*What kinds of things have you used an app or website to find with regard to your cancer – for example, information about treatment, nutrition, etc.*

*Can you give an example of a website or app that you like for finding information? Why do you like it?*

*If no, you don’t use apps or websites, why is that?*

# We know from what patients tell us, that brain cancer and its treatments cause symptoms and side effects that can be challenging and upsetting

# What symptoms/side effects have you or are you experiencing?

# *If yes, what have these been?*

# *Have any of these symptoms or problems been particularly burdensome – that is they have particularly impacted your ability to do day-to-day things or caused you more concern or distress?*

*How have you managed these or what help have you had to manage these?*

*Do you discuss symptoms or problems with a health professional during a hospital visit or appointment?*

*Some people tell us that they find it hard to describe the symptoms or problems they are having to members of your treating team. Is this something you experience?*

*If yes, who? Do you use a diary or app to record things?*

1. **If it was possible for you to keep a record of your symptoms or problems in an app or website would that be helpful to you?**

*If yes, why do you think that would be helpful?*

*How would you like to get feedback? For example, of graph or map of your symptoms*

*How often would it be helpful to have that kind of feedback?*

*How do you think you would use that information?*

*Do you think it would be helpful for the information you record to go to your treating team too?*

*If yes, why do you think that would be helpful?*

*Who should get your information?*

*Would you like to get feedback from the team on that information?*

*If yes, how and how often?*

*If no, is there something else you would find helpful- and, why?*

**For some symptoms or problems, such as tiredness or being unable to sleep, anxiety or fear of cancer recurrence, there are online programs that can help you cope with or manage these.**

1. **If you have or had any of these problems or symptoms, would you try an online program to manage them**?
   *If yes, can you tell me why? If no, can you tell me why?*

What would you hope to achieve from an online program (e.g. able to manage a problem yourself )?

*If yes, are there particular things you would need from the online program that would make you use it (e.g. – the accent of the person delivering it, how long it went on for, the ability to report back how you were going; having feedback from someone)*

1. **Have you had to use telehealth much since the pandemic started – or were you using it before? By telehealth we mean more than a telephone call, for example a video-consultation
   What’s your experience of telehealth been?**

*If it was possible to connect with your treating team for parts of your care and follow up via an online platform would you use it?*

*If yes, why would you be happy to do this- what benefits can you see?*

*If no, can you tell me why this wouldn’t work for you?*

**Conclusion:** *Is there anything else that you would like to tell me about your experiences or support needs before we finish today?*

**When we first spoke about the study, I mentioned the opportunity to be involved in a future workshop. Are you happy to be contacted to see whether you would be available to take part in a workshop over the next few weeks?**

**Yes**

**No**

**Thank you for sharing your experiences and for your time and interest in participating in the interview.**
